# Pressure Membrane FBG Sensor Realized by 3D Technology

**DOI:** 10.3390/s21155158

**Published:** 2021-07-30

**Authors:** Marcel Fajkus, Jan Nedoma, Radek Martinek, Michael Fridrich, Emil Bednar, Stanislav Zabka, Petr Zmij

**Affiliations:** 1 Department of Telecommunications, Faculty of Electrical Engineering and Computer Science, VSB-Technical University of Ostrava, 17. listopadu 2172/15, 708 33 Ostrava-Poruba, Czech Republic; jan.nedoma@vsb.cz (J.N.); michael.fridrich@vsb.cz (M.F.); emil.bednar.st@vsb.cz (E.B.); stanislav.zabka@vsb.cz (S.Z.); 2 Department of Cybernetics and Biomedical Engineering, Faculty of Electrical Engineering and Computer Science, VSB-Technical University of Ostrava, 17. listopadu 2172/15, 708 33 Ostrava-Poruba, Czech Republic; radek.martinek@vsb.cz; 3 Industrial Engineering—Brose Group, Prumyslovy Park 302, 742 21 Koprivnice, Czech Republic; petr.zmij@brose.com

**Keywords:** fiber Bragg grating, measurement, pressure, 3D technology

## Abstract

The publication describes the design, production, and practical verification of an alternative pressure sensor suitable for measuring the pressure of gas, based on a combination of fiber-optic technology and 3D printing methods. The created sensor uses FBG (Fiber Bragg Grating) suitably implemented on a movable membrane. The sensor is equipped with a reference FBG to compensate for the effect of ambient temperature on the pressure measurement. The sensor is characterized by its immunity to EM interference, electrical passivity at the measuring point, small size, and resistance to moisture and corrosion. The FBG pressure sensor has a pressure sensitivity of 9.086 pm/mbar in the range from 0 to 9 mbar with a correlation coefficient of 0.9982. The pressure measurement in the specified range shows an average measurement error of 0.049 mbar and a reproducibility parameter of 0.0269 ± 0.0135 mbar.

## 1. Introduction

The designed sensor, which is described in detail in this publication, is intended for measuring the pressure of liquids or gases. It can be used in many industries from industry 4.0, through automotive, aerospace, energy industries, and household to biomedicine. Pressure sensors are used to measure static or dynamic pressure, to measure their fluctuations, pulses, or turbulence. Typical applications are measuring the flow or level of liquids, pressure of liquids or gases in product pipelines, pumps and compressors, etc. Conventional pressure sensors use electrical components to set measuring ranges, reduce the effects of temperature characteristics, or to improve the linear characteristics of sensors. However, these sensors have a major disadvantage. They cannot be used in high-temperature, electromagnetic (EMI), or explosive environments [1].

The basic types of pressure sensors include resistance, capacitive, inductive, and resonant sensors. In the case of resistance sensors, a change in the resistance of the wire connected to the Wheatson bridge in the membrane is used, which is deformed by the action of pressure. Their disadvantage is temperature dependence. A typical example is a piezoresistive resistance sensor [2], which uses a monocrystalline phenomenon. Capacitive sensors [3], on the other hand, use the diaphragm as the capacitor electrode, the capacitance of which changes due to the action of pressure on the diaphragm. Inductive sensors [4] are constructed in a similar manner. Their membrane is made of ferromagnetic material and changes the inductance of the electrical connection due to the action of pressure. Resonant sensors, which comprise a string that oscillates due to electromagnetic excitation, work in a different way. The actual resonant frequency then depends on the mechanical tension of the string, which is attached to the membrane on one side.

Intelligent pressure sensors are also widespread, with electronic circuits that help in linearizing the conversion characteristics, setting the measuring range, reducing the temperature sensitivity, etc. However, the disadvantages include the impossibility of use in explosive and humid environments or in environments with high EM interference. These disadvantages are eliminated by fiber optic sensors that are based on the modulation of light passing through them. They are characterized by their immunity to EM interference, electrical passivity, small size and resistance to moisture, corrosion, etc.

In fiber optic sensors, two main principles are used to measure pressure. The first is based on measuring a change in position using a Fabry–Perot interferometer [5,6,7,8,9]. The disadvantage of this sensor is the high sensitivity to temperature changes due to thermal expansion. This shortcoming is eliminated by the application of the concept of a Fabry-Perot interferometer with an FBG sensor, which is used to measure temperature and compensate for its effect [10]. This sensor achieves a sensitivity of 501 nm/kPa in the range of 0 to 2 kPa.

The second category consists of sensors based on Bragg gratings [11,12,13,14,15,16,17,18,19,20,21,22] for the measurement of low and high pressures and vibrations. Zhang et al. [11] presented the pressure FBG sensor embedded in a polymer-filled metal cylinder with an opening on one side to enhance the pressure sensitivity. Based on this type of embedding, the authors declare that the sensitivity of the fractional change in the Bragg wavelength of experimental sensor is $-3.41\times 10^{-3}$ MPa${}^{-1}$ (about 1720 times higher than a bare fiber grating).

Liu et al. [12] developed an FBG pressure sensor partly shielded with a metal tube where the thermal-strain cross effect is avoided, and its pressure sensitivity is increased to $-2.44\times 10^{-3}$ MPa${}^{-1}$ (about 1200 times higher than a bare fiber grating). Vengal Rao et al. [13] described an FBG pressure sensor partly assembled with a thin metal diaphragm, where its pressure sensitivity was found to be $2.05\times 10^{-2}$ MPa${}^{-1}$ (about 10,350 times as that of a bare fiber grating).

Huang et al. [14] described an FBG pressure sensor based on a Bourdon tube with two FBGs bonded on its outside and inside surfaces, respectively (using two different FBGs, the sensitivity is enhanced; the temperature cross-sensitivity is also compensated). The measurement sensitivity is 1.414 pm/kPa in a range from 0 to 1 MPa (correlative coefficient reaches about 99 %).

Urban et al. [15] designed a pressure capsule with two sensing FBGs, where the first FBG senses pressure, and the second one was built inside the sensing capsule for parasitic temperature calibration. Results from tests showed that measuring precision is under 3.13% over a pressure range (0–1.8 bar) in the operating temperature range –20 ${}^{{}^{\circ}}$C to +70 ${}^{{}^{\circ}}$C.

Huang et al. in [16] presented interesting results in the form of a diaphragm-type FBG pressure sensor for measuring the static/dynamic pressure of a gas or liquid. The authors used two bare FBGs directly bonded on a circular diaphragm along with the radial direction. Results indicate the measurement sensitivity of 1.57 pm/kPa in a range from 0 to 1 MPa with a correlative coefficient of 99.9%. Pachava et al. in [17] showed a simple solution in the form of firmly fixing the FBG with a metal bellows structure. The pressure sensitivity of the sensor is found to be 90.6 pm/psi (about 4000 times higher than a bare fiber grating), and the linearity of the sensor reached a value of 99.86%.

Zeng et al. in [18] presented an in-pipe optical fiber sensor array based on the two FBG pressure sensors contained in a protective cable for transient pressure measurement and the implementation of the paired-IRF (Impulse Response Function) technique for leak detection in a laboratory copper pipeline. Another paper [19] by Wei et al. presented results in the form of a novel FBG sensor for measuring earth pressures in civil engineering. The sensor is based on the combination of a cantilever beam with two FBGs and a flexible membrane, and the sensitivity of the sensor is 0.104 kPa/με.

Guo et al. [20] presented promised results of a novel FBG sensor based on two FBGs. The sensor is composed of a circular elastic pressure-bearing membrane, and another part is the sensor enclosure and stainless steel top cover. Based on experimental results, it can be stated that, in the 50 MPa pressure range and a temperature range of 100–300 ${}^{{}^{\circ}}$C, the relative maximum error is only 4.6%. Aime et al. [21] described a solution based on the FBG and a glass fiber reinforced polymer membrane for use in aerodynamic applications with sensor sensitivities of 56.7 pm/kPa. The construction of the sensor is simple, but it is clever and consists of the FBG co-bonded on the surface of the membrane. An FBG pressure sensor encapsulated by a flat diaphragm structure described by Xiong et al. [22]. The sensor of the flat diaphragm is composed of a flat diaphragm, protective cylinder, and metallization FBG. Based on the experimental results, the authors declare that the sensitivity coefficient of the sensor is $2.3\times 10^{-3}$ MPa${}^{-1}$ for a measuring range of 0.5 MPa, and the linearity is 0.9720.

Another membrane sensor similar to our solution is designed for measuring water or oil pressure [23]. Fluid acting on the surface of the piston diaphragm in the axial direction causes tensile stress on the FBG. The authors declare the sensitivity of the sensor to be 7 nm/MPa. In another paper, the authors presented a specific encapsulation of the Bragg grating, thanks to which they achieved a high pressure sensitivity of $5.75\times 10^{-1}$ MPa${}^{-1}$ in the range of 0 to 160 kPa [24].

The study in this article focuses on the principle of the transverse measurement of the deflection of a membrane using 3D printing technology for encapsulation of the optical fiber Bragg grating and the implementation of the conversion of the effect of pressure on the measurement of deformation.

## 2. FBG Membrane Sensor

### 2.1. Fiber Bragg Grating

**Figure 1:**
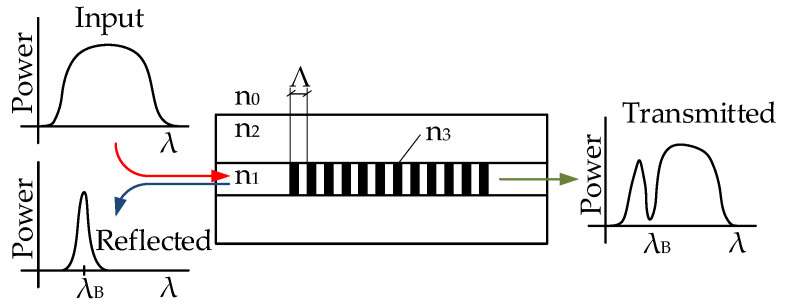
Principle of fiber optic Bragg grating.

The Bragg grating is formed by the periodic structure of changes in the refractive index in the core of an optical fiber. This structure consists of a repeating refractive index of the core $n_{1}$ and an increased refractive index, $n_{3}=n_{1}+\delta n$, which is generated by a UV laser incident on the optical fiber through a phase mask [25] or directly in the case of a point-by-point production [26]. The Bragg grating reflects a narrow spectral region of light, the central position of which, referred to as the Bragg wavelength $\lambda_{B}$, can be expressed by:

(1) $$\lambda_{B}=2n_{eff}\Lambda ,$$

where $n_{eff}$ is the effective refractive index of the FBG structure, and Λ is the period of refractive index change. The structure of the Bragg grating is shown in Figure 1. If we introduce light from a broad-spectrum radiation source into an optical fiber with FBG, then the narrow spectral region corresponding to Equation (1) is reflected, and the remaining spectral part passes through the structure.

The Bragg grating is used as a sensing element for measuring a number of quantities. The principle in sensors’ applications is based on the change of the Bragg wavelength due to the change of geometric and optical properties of the FBG structure due to the action of an external quantity. The shift of the Bragg wavelength can be written as the equation:

(2) $$\frac{\Delta \lambda_{B}}{\lambda_{B}}=\left(1-P_{e}\right)\epsilon +\left(\alpha_{\Lambda }+\alpha_{n}\right)\Delta T,$$

where $P_{e}$ is the elasto-optic coefficient, ϵ is the size of axial deformation, $\alpha_{\Lambda }$ is the coefficient of thermal expansion, $\alpha_{n}$ is the thermo-optic coefficient, and ΔT is the change in temperature.

### 2.2. Membrane-Based FBG Pressure Sensor

**Figure 2:**
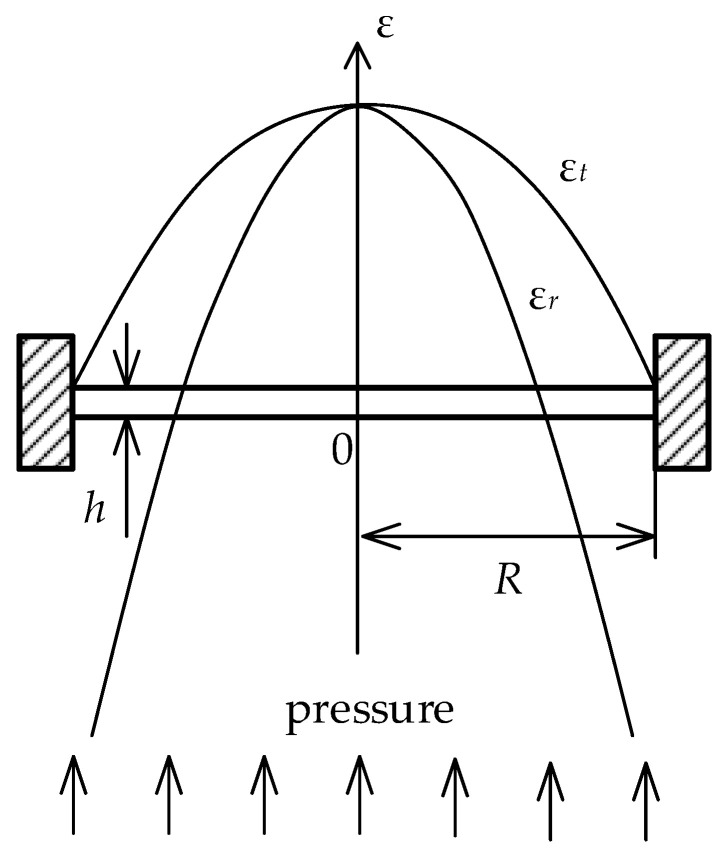
Circular membrane with indication of radial and tangential components of deformation caused by pressure.

The principle of operation of the presented pressure sensor formed by a circular membrane is indicated in Figure 2. The figure shows a membrane with a radius *R* and a thickness of the membrane *h*, which is anchored between two points. If pressure is applied to the diaphragm, its force will cause the diaphragm to deflect.

With respect to the elementary Hooke’s law, dealing with the theory of small deformations, the deflection of the membrane can be defined by a radial component $\epsilon_{r}$ and a tangential component $\epsilon_{t}$. Both components have the same value in the center of the membrane that can be expressed by the equation:

(3) $$\epsilon_{c}=\frac{3pR^{2}\left(1-\mu^{2}\right)}{8Eh^{2}},$$

where *p* is the pressure affecting the membrane, *E* is Young’s modulus of elasticity and μ is Poisson’s ratio of membrane material, *h* is the thickness of the membrane, and *R* is its diameter.

### 2.3. Construction of a Membrane-Based FBG Pressure Sensor

**Figure 3:**
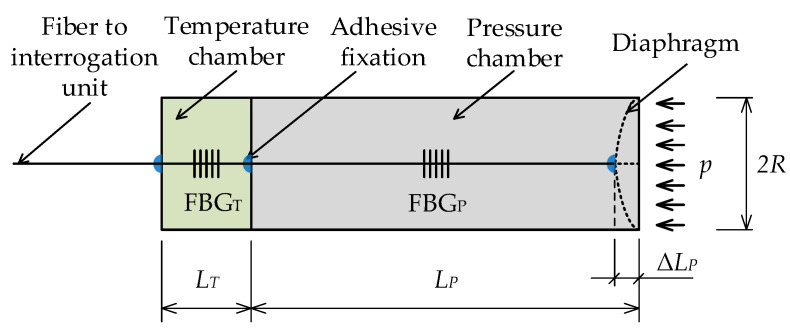
Construction of a pressure membrane of the FBG pressure sensor with temperature compensation $FBG_{T}$.

To measure pressure, the measurement of the deflection of the membrane using an optical fiber Bragg grating is used. The construction of the sensor consists of a cylinder, which is provided with a membrane on one side. This membrane cambers axially into the cylinder due to external pressure (Figure 3). The optical fiber with the Bragg grating *FBG*${}_{P}$ is fixed on a moving membrane and on the other side on the base of the cylinder in the pressure chamber with length $L_{P}$. When being affixed, the optical fiber is pre-tensioned so that it can be shortened during the measurement by $\Delta L_{P}$. The applied pressure on the membrane causes it to bend and shorten the optical fiber. As a result, the reflected Bragg wavelength shifts toward lower wavelengths. The temperature effect is compensated by the second Bragg grating *FBG*${}_{T}$, which is located in the temperature chamber.

The sensor housing is composed of three parts. The first part consists of the cylinder with the pressure chamber, and the second part consists of the cylinder of the temperature chamber, which was made without one wall. The third part consists of the pressure sensing diaphragm. All parts were printed on a 3D printer using Fused Deposition Modeling (FDM) technology. All the components were printed from ABS (Acrylonitrile Butadiene Styrene) filament with an elasticity modulus of 2174 ± 285 MPa, with a layer height of 0.2 mm and a filler of 60%.

The dimensions of the sensor are as follows: the radius of the housing is 19 mm, the length of the pressure chamber is 100 mm, the length of the temperature chamber is 30 mm, the wall thickness of the housing is 4 mm, and the wall thickness between the temperature and pressure chambers is 7 mm. The membrane itself was printed separately, and its thickness is 0.6 mm. The membrane was attached to the housing with a two-component epoxy adhesive. The optical fiber (G.657.D in primary acrylate protection) is affixed to the membrane, to the wall in the pressure chamber, and the temperature chamber in the same way.

The detection principle is based on the fact that the increasing pressure shortens the optical fiber stretched in the pressure chamber, and, for this reason, the optical fiber was preloaded during installation from an unloaded wavelength of 1543.847 nm by 1.029 nm, which corresponds to a deformation of about 800 μstrain.

An optical fiber with a temperature-compensating Bragg grating $FBG_{T}$ with a reflectance spectrum at a wavelength of 1521.357 nm was placed in the temperature chamber. To prevent the fiber from moving and affecting the measurements, the fiber was preloaded to a wavelength of 1521.877 nm and fixed at both ends of the chamber with a two-component epoxy adhesive. Both fiber Bragg gratings used were manufactured in single-mode G.657.D standard optical fiber with a primary polyimide coating, which exhibits a tighter bond between the fiber’s glass cladding and the primary coating, which is important in strain measurements. The temperature Bragg grating $FBG_{T}$ and the pressure Bragg grating $FBG_{P}$ had spectral width at a half maximum (FWHM) of 203 pm and 226 pm, respectively, and reflectances of 87% and 85%, respectively.

**Figure 4:**
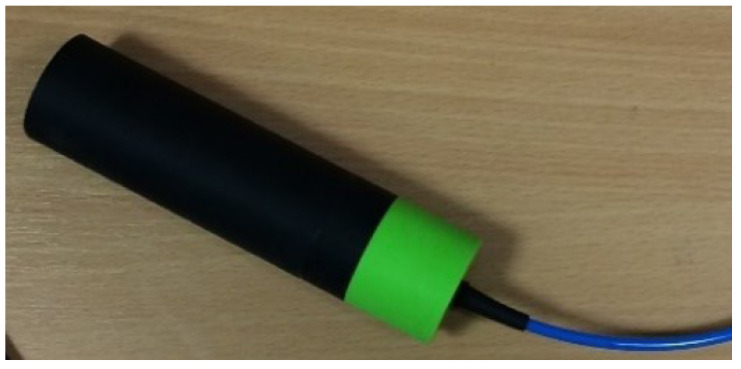
Execution of a membrane pressure sensor.

The resulting pressure sensor corresponding to the diagram in Figure 3 is shown in the following Figure 4. The pressure chamber is printed with a black, and temperature chamber with a green, ABS type filament.

## 3. Pressure Measurement Using an FBG Membrane Sensor

### 3.1. Temperature Compensation of the Pressure Sensor

The pressure sensor is designed to compensate for ambient temperature fluctuations that affect both fiber optic Bragg gratings. To determine the temperature sensitivities of the Bragg gratings, the pressure sensor was placed in a temperature box in which the temperature was set from −20 to 60 ${}^{{}^{\circ}}$C. The temperature and pressure Bragg grating exhibit temperature dependence of $k_{1T}=14.2$ ${}^{{}^{\circ}}$C and $k_{2T}=17.2$ ${}^{{}^{\circ}}$C, respectively.

**Figure 5:**
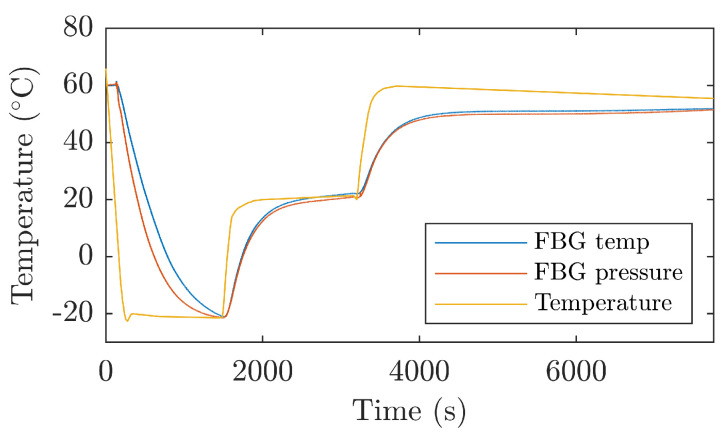
Temperature measurement using temperature $FBG_{T}$ and pressure $FBG_{P}$ Bragg grating in the temperature box.

Figure 5 shows the experimental temperature measurement in the temperature box with the reference temperature sensor (orange line), the calculated temperature from $FBG_{T}$, and the temperature from $FBG_{P}$.

The measurements show that the Bragg grids exhibit some hysteresis in temperature measurements. This hysteresis is evident for large and rapid changes in ambient temperature. In real conditions, where such rapid temperature changes do not arise, this hysteresis does not appear. Based on the results of long-term measurements in the thermal box, fiber Bragg gratings were operational at 60 ${}^{{}^{\circ}}$C for four hours. When the temperature was further increased to 70 ${}^{{}^{\circ}}$C, the optical fiber fixation to the sensor housing was loosened after 20 mins, causing the correct pressure measurement in the pressure chamber to cease. This loosening was caused by the type of adhesive used. On the basis of the experiments performed, a working range from −20 ${}^{{}^{\circ}}$C to 40 ${}^{{}^{\circ}}$C can be defined, and further with short-term temperatures up to 60 ${}^{{}^{\circ}}$C. The increase of the temperature working range can be further increased by using a suitable adhesive whose strength will be guaranteed even at higher temperatures.

Temperature compensation is performed by evaluating the signal from both Bragg gratings simultaneously. Based on the known temperature coefficient of the Bragg grating $FBG_{T}$, the temperature change is determined according to the relation:

(4) $$\Delta T=\frac{\Delta \lambda_{BT}}{k_{1T}},$$

where $\Delta \lambda_{BT}$ is the change in the Bragg wavelength of the Bragg grating $FBG_{T}$ due to temperature change and $k_{1T}$ is its temperature coefficient. This temperature change causes a change in the Bragg wavelength of $FBG_{P}$ of size $k_{2T}\Delta T$, where $k_{2T}$ is the temperature sensitivity of the pressure Bragg lattice of $FBG_{P}$. The temperature-compensated measured pressure acting on the pressure sensor can be determined by equation:

(5) $$\Delta P=\frac{1}{k_{2P}}\left(\Delta \lambda_{BP}-\frac{k_{2T}}{k_{1T}}\Delta \lambda_{BT}\right)$$

where $k_{2P}$ is the pressure sensitivity coefficient of the Bragg grating $FBG_{P}$.

### 3.2. Sensitivity of the Sensor to the Membrane Load

**Figure 6:**
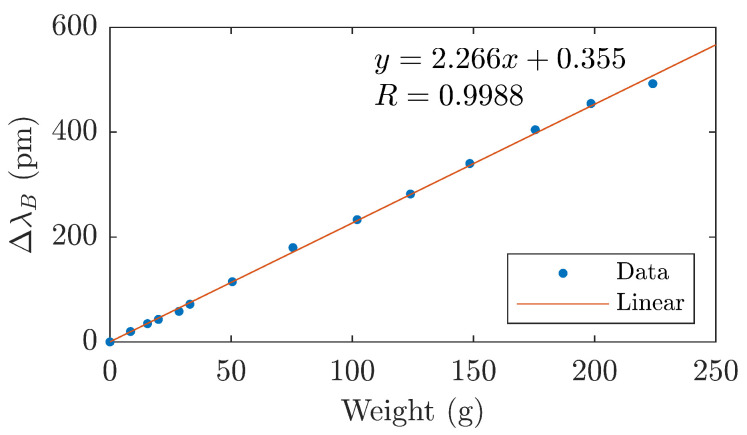
Pressure sensor calibration curve, Bragg wavelength dependence on membrane load.

In the first phase, the membrane load was measured with weights starting at 8 g. The response of the pressure sensor, i.e., the Bragg grating monitoring the deflection of the membrane, is shown in the figure below (Figure 6). The dependence is shown up to a weight of 223 g, which represents the yield strength of a membrane made with 3D printing—see the previous chapter. When this load is exceeded, the elastic deformation changes to plastic behavior. The change of the Bragg wavelength is linearly dependent on the mass of the weight with the parameter R-square 0.9988, and the sensitivity coefficient of the pressure sensor to the weight of the diaphragm load is 2.266 pm/g.

### 3.3. Experimental Work Station for Pressure Measurement

**Figure 7:**
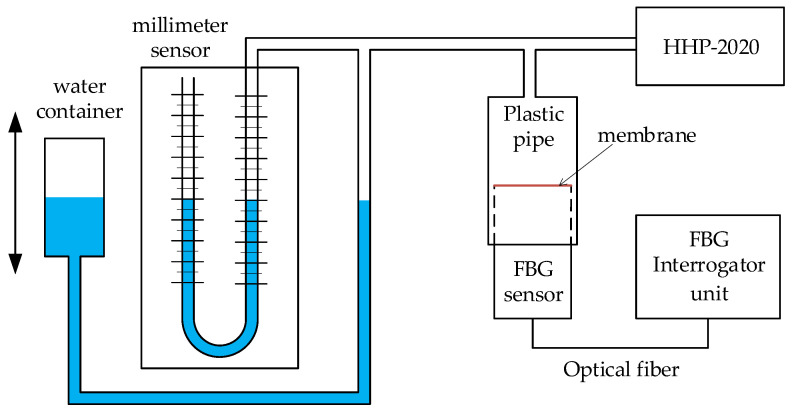
Diagram of experimental pressure measurement using a millimeter sensor, digital manometer HHP-2020, and FBG sensors.

Experimental verification of the sensor’s functionality was performed using a reference digital manometer HHP-2020 (Omega Engineering, Norwalk, CT, USA) and a classic column millimeter pressure sensor. The wiring diagram is shown in Figure 7.

The crucial element for setting the pressure was the water container, which was connected by a hose to a millimeter pressure sensor. A branch from this tube was connected to a digital manometer and to a plastic pipe. An FBG sensor was inserted into this plastic tube from the bottom. To prevent pressure loss, a seal was used between the plastic tube and the FBG sensor itself. The FBG sensor was connected to the evaluation unit—an FBGuard 1550 Fast (Safibra, Prague, Czech Republic).

The above experimental pressure measurement diagram was used in the calibration phase of the diaphragm sensor and in the verification of functionality in cyclic pressure measurement—see the following chapters.

### 3.4. Sensor Calibration

At the beginning of the calibration measurement, the water container was placed at such a height that the fluid level in this vessel was at the same level as the fluid in the millimeter sensor. Subsequently, the fluid bottle was lifted, increasing the pressure in the tubing. This pressure is the same everywhere, so its value is measured by a millimeter sensor, digital manometer, and FBG sensor.

**Figure 8:**
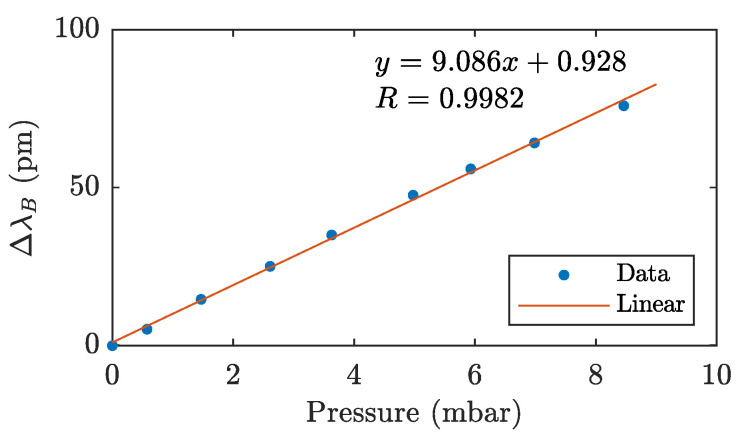
Calibration of the FBG pressure sensor.

During the measurement, the pressure was increased from 0 mbar to 8.5 mbar. The measured points of the calibration curve, and thus the dependence of the change in the Bragg wavelength on the applied pressure, are shown in Figure 8. Here, data from a millimeter sensor with a sensitivity of 0.098 mbar/mm scale were used for the pressure reference value.

A linear approximation with a sensitivity coefficient of 9.086 pm/mbar and an R-square parameter of 0.9982 was compiled from the measured data. Based on the calibration measurement, a linear equation was compiled to calculate the pressure based on the change in the Bragg wavelength of the membrane FBG sensor:

(6) $$p=\frac{\Delta \lambda_{B}}{k_{2P}},$$

where $k_{2P}$ is the pressure sensitivity coefficient of $FBG_{P}$ with a value of 9.086 pm/mbar.

Previous measurements show that, with this type of sensor, it is possible to measure not only the pressure *P* but also the weight *m*. There is a simple conversion between the pressure and the weight acting on an area. The force acting on the diaphragm is given by $F=ma_{g}$, where *m* is the load weight and $a_{g}$ is the gravitational acceleration, whose theoretical mean value at the surface of the earth is 9.823 m/s${}^{2}$. The pressure generated on the diaphragm is then given by P=F/S, where *S* is the area of the diaphragm. To convert the weight and pressure, the simple relationship $P=ma_{g}/S$ can be derived; then, a mass of 50 g will put pressure on the membrane of 433.07 Pa, which is equivalent to 4.33 mbar. Since the pressure-induced deflection of the diaphragm is composed of a radial and tangential component, the general relationships given here do not apply. To calculate the weight and pressure, respectively, it is necessary to use the relationships given in Figure 6 (weight measurement) and Figure 8 (pressure measurement).

### 3.5. Cyclical Pressure Measurement

**Figure 9:**
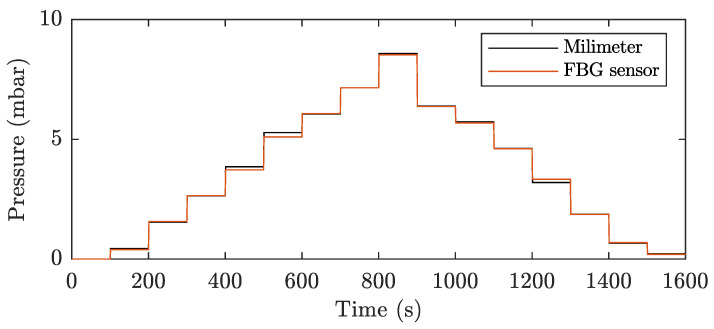
Comparison of one pressure measurement cycle with a millimeter reference pressure gauge and diaphragm FBG sensor.

Figure 9 shows a detail of one cycle (out of a total of 50 performed measurement cycles) of pressure measurement, where the pressure was gradually increased from the initial value of 0 mbar to a maximum value of 8.526 mbar and then gradually reduced to zero value (the final value pressure was 0.196 mbar, see Table 1).

Table 1 shows the measured data from a stated pressure measurement cycle from 0 mbar to a maximum pressure of 8.526 mbar and a subsequent decrease to 0 mbar. The table shows the values measured with a column and digital pressure sensor. For the FBG sensor, the measured changes in the Bragg wavelength and converted pressure according to Equation (4) are given. The last columns show the absolute and relative error of the FBG sensor relative to the reference column pressure gauge.

The results of the total measurement of 50 cycles performed show that pressure measurement using a membrane FBG sensor reaches a maximum error of 0.194 mbar at a pressure of 5.136 mbar, which represents a relative error of 3.777%. The FBG sensor shows a large relative measurement error at very low pressures below 1 mbar, where the relative error reaches values greater than 10%. The proposed membrane sensor has an average measurement error of 0.049 mbar and a reproducibility of measurement (σRepeatability) of 0.0269 ± 0.0135 over the entire measurement range, i.e., from 0 to approx. 9 mbar.

**Figure 10:**
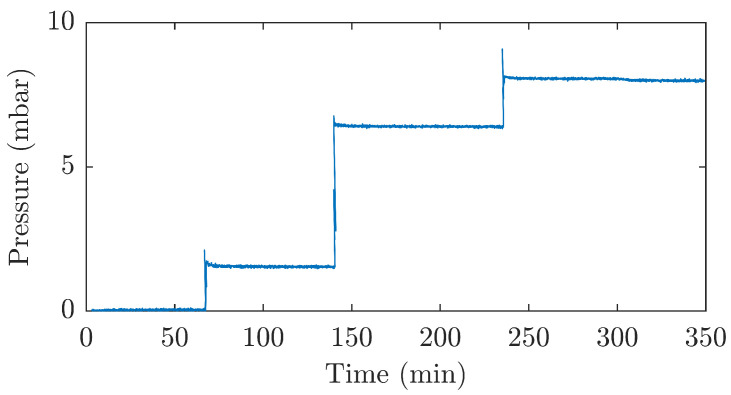
Pressure sensor response to long-term constant pressure loading.

An important parameter of the designed sensor is its ability to correctly measure static or unchanging pressure over a long time interval. For this reason, measurements were made in which the sensor was loaded with a constant pressure for a long period of time. The measured data are shown in Figure 10. During the measurement, the sensor was loaded with a pressure of 1.6 mbar for 73 min, a pressure of 6.5 mbar for 94 min and, in the last step, a pressure of 8.1 mbar for 113 min.

**Figure 11:**
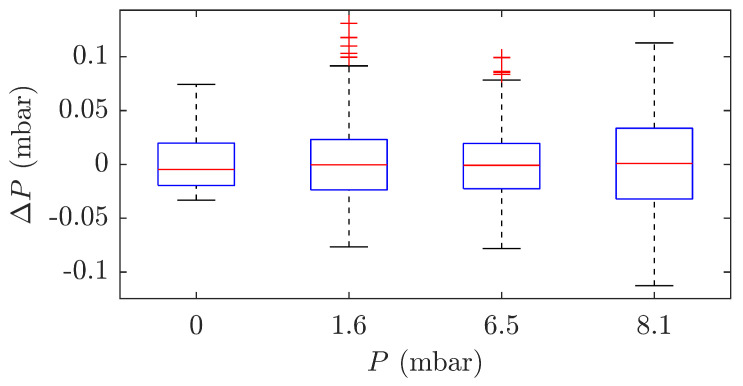
Static pressure measurement stability analysis.

Figure 11 shows the variance of the measured data in each sensor load condition. For better visualization, the mean value is removed from the measured data. The results show that the sensor is capable of measuring static pressure with a deviation of ±0.1 mbar. In the range of 0 to 10 mbar, this represents a deviation of 10% for pressures close to 0 and 1% for pressures close to 10 mbar.

## 4. Conclusions

The authors of this publication describe a comprehensive design and the verification of a pressure sensor using fiber-optic technology (FBG) and 3D printing technology. The principle of operation of the sensor is based on the appropriate implementation of FBG on the moving membrane, and an alternative sensor can be used to measure the pressure of liquids or gases. Thanks to the use of purely fiber technology (conventional optical fibers and FBG) and the 3D printing method, the sensor is characterized by its low cost (the production cost is about $150), immunity to EM interference, electrical passivity at the measuring point, and small size and resistance to moisture and corrosion. Corrosion resistance is ensured by the use of non-metallic parts of the sensor. Maximizing moisture resistance was ensured by the use of optical fiber and the appropriate choice of filament used to make the housing of the pressure sensor. The FBG pressure sensor has a pressure sensitivity of 9.086 pm/mbar in the range from 0 to 9 mbar with a correlation coefficient of 0.9982. Based on the practical verification of the pressure measurement, the sensor is characterized in the above range by an average measurement error of 0.049 mbar, and the reproducibility parameter has a value of 0.0269 ± 0.0135 mbar.

## Figures and Tables

**Table 1:** Summary of pressure measurement with a millimeter column sensor, digital manometer HHP-2020, and membrane FBG sensor.

	Milimeter Pressure Sensor		HHP-2020	FBG Sensor		Absolute Error FBG	Relative Error FBG
ID	(mm)	(mbar)	(mbar)	$\Delta \lambda_{B}$(pm)	*p* (mbar)	*p* (mbar)	*p* (%)
1	0	0	0	0	0	0	0
2	4	0.392	0.425	4	0.440	0.048	12.306
3	16	1.568	1.590	14	1.541	−0.027	−1.733
4	27	2.646	2.620	24	2.641	−0.005	−0.173
5	38	3.724	3.786	35	3.852	0.128	3.439
6	52	5.096	4.990	48	5.283	0.187	3.667
7	62	6.076	5.940	55	6.053	−0.023	−0.374
8	73	7.154	7.050	65	7.154	0	−0.002
9	87	8.526	8.470	78	8.585	0.059	0.688
10	65	6.370	6.430	58	6.383	0.013	0.211
11	58	5.684	5.600	52	5.723	0.039	0.688
12	47	4.606	4.600	42	4.622	0.016	0.358
13	34	3.332	3.300	29	3.192	−0.140	−4.210
14	19	1.862	1.800	17	1.871	0.009	0.484
15	7	0.686	0.700	6	0.660	−0.026	−3.738
16	2	0.196	0.180	2	0.220	0.024	12.306

## Data Availability

Not applicable.
